# A rare ocular complication of septicemia: a case series report and literature review

**DOI:** 10.1186/s12879-023-08489-1

**Published:** 2023-08-09

**Authors:** Tang Xu-yuan, Li Hui-yan

**Affiliations:** Department of Ophthalmology, The First Affiliated Hospital, School of Medicine, Zhejiang University, Hangzhou, China

**Keywords:** Septicemia, Orbital Cellulitis, Hematogenous, Immunocompromised

## Abstract

**Background:**

Septicemia that leads to ocular involvement mostly presents as endophthalmitis or panophthalmitis. Contrarily, septicemia without intraocular involvement, known as hematogenous orbital cellulitis (HOC), involves only the orbit and is an extremely rare complication of septicemia and a rare type of orbital cellulitis.

**Case presentation:**

Four male patients with septicemia presented with orbital involvement without intraocular infection were described in this study. They were 22 (case 1), 15 (case 2), 79 (case 3), and 30 (case 4) years old, with a mean age of 29.75 years. All patients were immunocompromised except for case 2. Cases 1 and 3 had a history of steroid use, whereas case 4 was in a post-chemotherapy myelosuppression phase. Septicemia in case 1 was community-acquired, cases 3 and 4 were hospital-acquired, and case 2 was secondary to acne squeezing. Blood cultures from cases 1, 2, and 3 were positive for *Candida albicans*, methicillin-resistant *Staphylococcus aureus*, and *Klebsiella pneumoniae*, respectively. Case 4 had negative cultures; however, next-generation sequencing reported the presence of *Enterococcus faecalis* and *Rhizopus oryzae.* Case 1 had right eye involvement, and both eyes were involved in the other three cases. According to Chandler’s classification, case 1 was type 2, case 2 was type 2 (OD) and type 4 (OS), and cases 3 and 4 were type 1 orbital infections. All patients had eyelids erythema, and cases 1 and 2 had mildly decreased visual acuity, proptosis, and painful and restricted ocular motility. Hospital stays ranged from 13 to 43 days (mean, 24 days). All patients received systemic antibiotic therapy based on drug sensitivity and next-generation sequencing results, in combination with multidisciplinary treatment, resulting in complete recovery of ocular and systemic signs and symptoms; no ocular surgical interventions were performed. Extraocular muscle palsy was the last symptom to resolve.

**Conclusion:**

HOC is predominantly seen in immunocompromised individuals with a high proportion of hospital-acquired infections and positive cultures for pathogens. Infection control using systemic antibiotics targeted at the causative organism guarantees a favorable prognosis.

## Background

Orbital cellulitis (OC) is a common orbital disease that typically arises from the direct spread of nearby infection and is more prevalent in children than adults [1, 2]. Hematogenous orbital cellulitis (HOC), an extremely rare type of orbital cellulitis, is caused by infection spread from a distant source via blood transfer to the orbit without intraocular involvement, and is an uncommon ocular complication of septicemia that mostly presents as endophthalmitis or panophthalmitis. HOC has different pathogenic profiles and clinical manifestations than contiguous spreading OC; nevertheless, very few studies have been reported on this type of OC. Herein, we present four cases of HOC involving five rare causative organisms for OC.

## Case presentation

### Case 1

**Fig. 1:**
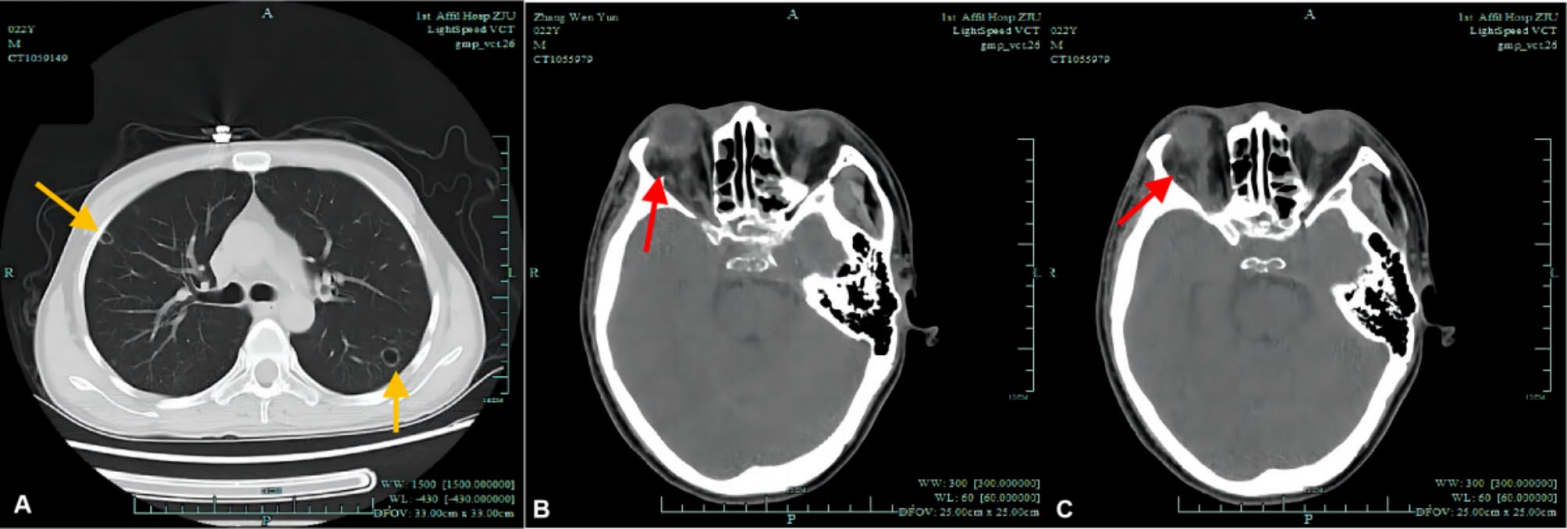
Lung and orbital CTs of case 1 **(A)** On admission, multiple cavernous nodules were detected in both lungs, mainly concentrated in the periphery of the lungs (yellow arrows). **(B**, **C)**: Right eye protrusion with infection mainly located in the lateral **(B)** and superior(C) parts of the right orbit (red arrows)

A 22-year-old man experienced cough, sticky sputum, and high temperature for 3 days and then developed right eyelid swelling, followed by right ear pus discharge and ocular pain. Intravenous amoxicillin was administered at a local clinic with no relief; therefore, he was referred to our emergency room and admitted to the Infectious Ward. Apart from a 14-year steroid medication history for non-infectious skin diseases, no other significant medical history was reported. On admission, he was malaise with a stiff neck and prominent breath sounds. Additionally, ophthalmologic examination (OE) revealed better visual acuity (VA) than finger counting (FC) at 1 m, fixed and protruding right eye with swollen and red eyelids, conjunctival infection and hemorrhage, sensitive pupillary reflex, but no lens and fundus abnormalities. The left eye was unremarkable. Lung computerized tomography (CT) revealed cavitated nodules in both lungs (Fig. 1A); orbital CT showed proptosis, infectious lesions in the upper and lateral sides of the retrobulbar orbit, mastoiditis, maxillary and ethmoid sinusitis, and right otitis (Fig. 1B, C). No orbital abscess was present, and according to Chandler’s classification of orbital infection, the patient had a type 2 infection [3]. Magnetic resonance imaging (MRI) could not be performed completely. Lumbar puncture (LP) revealed a high intracranial pressure (ICP) of 380 mmH_2_O with clear cerebrospinal fluid (CSF). *Candida albicans* (*C. albicans*) were isolated by culturing blood samples using the BacT/ALERT® 3D automated microbiological testing system (bio-Mérieux, France). Sputum and ear pus samples also yielded *C. albicans*, but CSF showed negative result. All samples were also cultured anaerobically using BacT/ALERT® FN anaerobic medium.The same culture methods were used for the next three cases.In addition to efforts for lowering ICP and phlegm treatment, intravenous meropenem (MEM) was administered for 14 days, followed by fluconazole (FCA) and moxifloxacin (MXF) for 12 and 8 days, respectively. No ocular invasive procedure was carried out. Table 1 lists the peak values of the inflammatory indicators during the patient’s hospitalization. Fifteen days after admission, when all signs and symptoms except eyelid swelling and 10° of esotropia resolved and the laboratory tests came back normal, the patient was discharged. The last follow-up visit at 4 months showed complete resolution of systemic and ocular infection signs, and the VA was 20/20 in both eyes. Table 1 shows the values of inflammatory indicators at discharge, and Table 2 summarizes the clinical overview of all four patients.

**Table 1:** Inflammatory indicators of the four HOC cases

No.	Indicators	T	WBC	NEUT	CRP	ESR	PCT
		(℃)	(×10E9/L)	(%)	(mg/L)	(mm/1 h)	(ng/ml)
Case 1	Peak	38.7	11.2	85.8	345.3	75	0.21
	Discharge	36.7	4.8	70.1	8.5	65	NA
Case 2	Peak	39.2	15.2	93.6	149.8	51	1.7
	Discharge	36.5	3.7	44.9	0.32	5	0.04
Case 3	Peak	40.3	20.5	97.3	125.01	6	56.02
	Discharge	36.8	9.0	79.7	52.63	NA	5.25
Case 4*	Peak	39.9	73.0	11.7	252.9	NA	0.21
	Discharge	36.9	3.5	73.0	44.9	NA	0.09

T, temperature; WBC, white blood cell; NEUT, neutrophilic granulocyte; CRP, C-reactive protein; ESR, Erythrocyte Sedimentation Rate; PCT, procalcitonin; NA, not applicable;

* This case had acute myeloid leukemia and received chemotherapy during hospitalization.

**Table 2:** Overview of the four HOC cases

No.	Age/SEX/ laterality	Ocular Clinical presentation	systemic Clinical presentation	Source of Infection	Immunosuppressive/cause	Culture /NGS	Chandler Classification	ABx (Duration, day)	CNS involvement /ICP (mmH_2_O)	Length of stay (days)	Initial BCVA	Final BCVA	Follow-up (month)
1	22/M/OD	Pain; proptosis; lid swelling and erythema; subconjunctival hemorrhage; conjunctival injection; limitation of OM;	high fever; Cough; Sputum; stiff neck; malaise;	*C. albicans* pneumonia induced *C. albicans* septicemia	(+)/GC medication for Skin disease	Sputum: *C. albicans* Ear pus: *C. albicans* Blood: *C. albicans* CSF:(-)	Type 2	MEM (14) FCA (12) MXF (8)	(+)/380	15	>VOU FC/1m (bedside examination)	VOU 20/20	4
2	15/M/OU	Ptosis and proptosis; lid swelling and erythema; conjunctival injection; conjunctiva pus (OS); limitation of OM;	high fever; malaise; Headache;	Forehead pustule-induced MRSA septicemia	(-)/NA	Blood: MRSA CSF: MRSA Sputum:(-)	OD: Type 2 OS: Type 4	TZP (2) LZD (23) DAP (12)	(+)/270	43	VOD 20/25 VOS 20/60	VOU 20/25	28
3	79/M/OU	Ophthalmoplegia (TAO); proptosis (TAO); lid swelling and erythema;	high fever; cough; Sputum;	*K. pneumoniae* pneumonia induced *K. pneumoniae* septicemia	(+)/GC medication for TAO	Blood: *K. pneumoniae* Sputum: *K. pneumoniae*	OU: Type 1	CFX (1) IMP (7)	(-)/NA	13	VOU FC/30 cm	VOD 20/50 VOS 20/40	7
4	30/M/OU	Lids tenderness; lid swelling and erythema; eyelid bruises; auricle swelling and tenderness; retinal hemorrhage (OD, AML);	high fever; cough;	*E. faecalis* septicemia *R. oryzae* septicemia	(+)/Chemotherapy for AML	Blood: (-) NGS: *E. faecalis*; *R. oryzae*;	OU: Type 1	CXM (8) MEM (5) CFP (12) VAN (10) TGC (6) POS (5 m) VRC (6) L-AMB (7)	(-)/NA	25	>VOU FC/1m (bedside examination)	VOU 20/25	6

HOC, Hematogenous orbital cellulitis; OM, Ocular motility; ABx, Antibiotics; CNS, Central nervous system; ICP, Intracranial pressure; BCVA, Best corrected visual acuity; OD, right eye; M, Male; OM, Ocular motility; GC, Glucocorticoids; CSF, Cerebrospinal fluid; MEM, Meropenem; FCA, Fluconazole; MXF, Moxifloxacin; OU, Both eyes; FC, Finger counting; NA, Not applicable; MRSA, *Methicillin-resistant Staphylococcus aureus*; TZP, Piperacillin-tazobactam; LZD, Linezolid; DAP, Daptomycin; OS, Left eye; TAO, Thyroid-associated ophthalmopathy; CFX, Cefoxitin; IMP, Imipenem; AML, Acute myeloid leukemia; CXM, Cefuroxime; CFP, Cefoperazone; VAN, Vancomycin; TGC, Tigecycline; POS, Posaconazole; VRC, Voriconazole; L-AMB, Liposomal amphotericin B

### Case 2

**Fig. 2:**
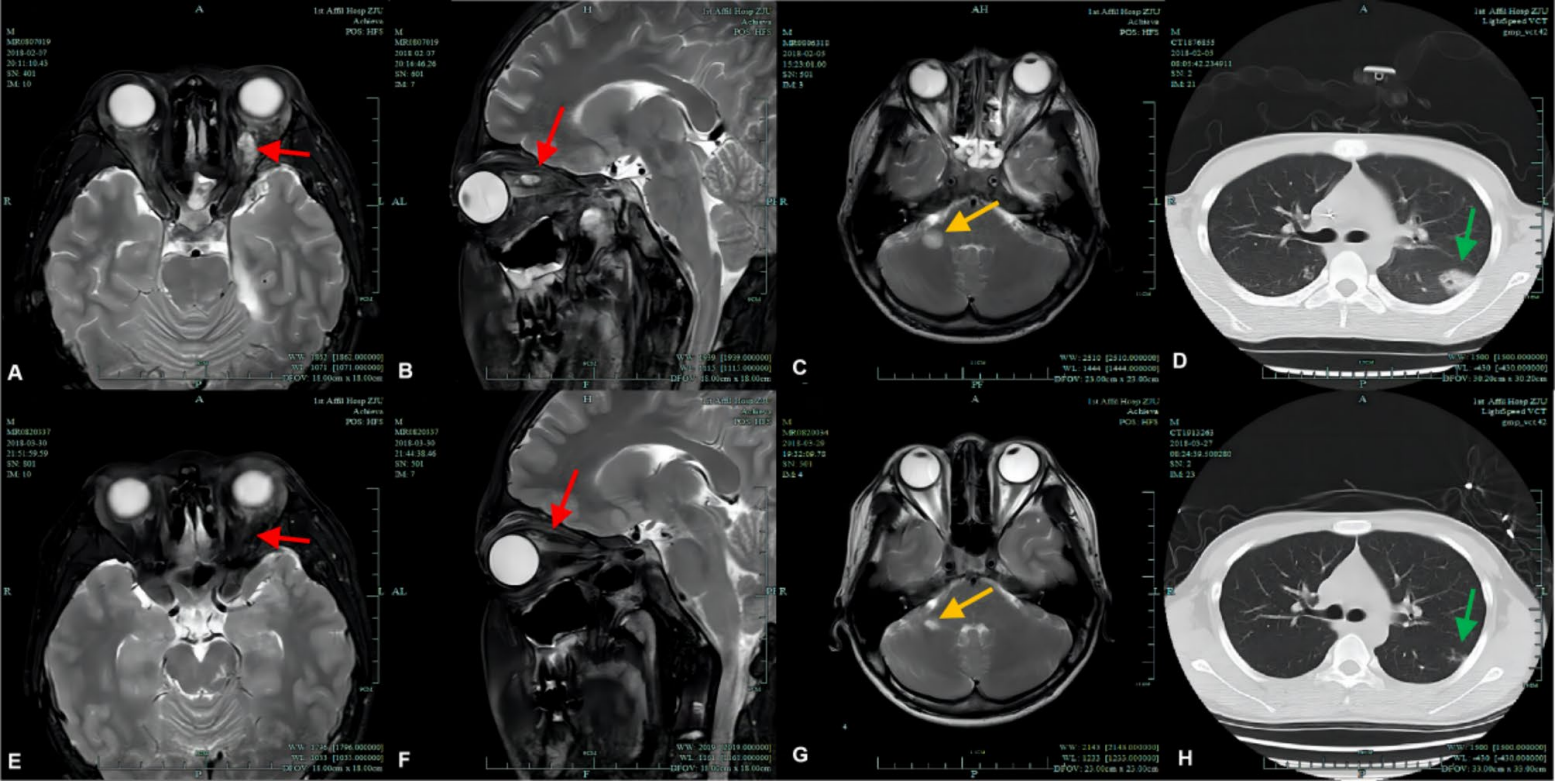
Orbital MRI, head MRI, and lung CT of case 2 (**A**, **B**, **C**, and **D**) were examined on early admission and (**E**, **F**, **G**, and **H**) were re-examined 50 days later before discharge. **(D)** Lung CT showing bilateral lung inflammation, which was more severe on the left side (green arrows). (C) Head MRI (T2, coronal) showing brain abscess formation (yellow arrows). (**A,B**) Orbit MRI (T2, coronal; T2, sagittal) showing bilateral orbital inflammation and abscess formation in the upper left orbit (red arrows). (**E**, **F**, **G**, and **H**) Follow-up MRIs and CTs showing that the inflammation and abscesses in the lungs, cerebrum, and orbits had mostly resolved before discharge

A 15-year-old boy developed a fever and headache 2 days after squeezing a pimple on his forehead. He was admitted to a local hospital with skin furuncle, septicemia, purulent meningitis, and bilateral OC, for which he was administered amoxicillin (AM), vancomycin (VAN), ceftazidime (CAZ), and linezolid (LZD); however, his high fever (> 39 °C) persisted. He was then transferred to our hospital’s infection unit. On admission, he had malaise and a VA of 20/25 in the right eye and 20/60 in the left eye. Both eyes showed eyelids erythema, tenderness, and conjunctival infection, and all these symptoms were more severe on the left side. OM was initially normal and intraocular infection was absent. The patient’s lung CT revealed several infectious lesions in both lungs and slight pleural effusion (Fig. 2D). Cranial MRI revealed several abscesses (Fig. 2C). LP revealed CSF turbidity with an ICP of 270 mmH_2_O. *Staphylococcus aureus (SA)* was isolated from blood and CSF samples two days later and antibiotic susceptibility testing was performed using the VITEK 2 Compact system (bio-Mérieux, France). It was identified as *Methicillin-resistant Staphylococcus aureus (MRSA)* by the Cefoxitin screening test and oxacillin MIC determination. On the third day, the boy developed a conjunctive purulent patch in his left eye with ptosis and proptosis of the left eye and limited abduction of both eyes. Ocular B-ultrasound revealed a left orbital abscess that was confirmed using orbital MRI, which further revealed bilateral maxillary sinusitis, sigmoid sinusitis, sphenoid sinusitis, and left mastoiditis (Fig. 2A,B). Prior to microbiological testing, the patient was administered piperacillin-tazobactam (TZP) for 2 days. Subsequent to the results of the microbiological testing, LZD and daptomycin (DAP) were administered for 23 and 12 days, respectively, in combination with supportive therapies, such as hepatoprotection and gastric protection, ICP reduction, expectorant administration, and nutritional support. The symptoms and signs resolved and the inflammatory indicators returned to normal after 43 days of hospital stay (Table 1) with mild redness and swelling of the eyelids and limited abduction of the left eye. Upon discharge, repeated CT of the lungs, orbit, and head revealed a significant reduction and resolution of the abscesses (Fig. 2E F,2G,2 H); however, the VA remained the same as that at admission. No surgical intervention was performed. The patient was followed up for 28 months. By the 3rd month, his VA had improved to 20/25 bilaterally, and the swelling in both eyelids had subsided. By the 14th month, his diplopia and left lateral rectus palsy had resolved completely (Table 2).

### Case 3

**Fig. 3:**
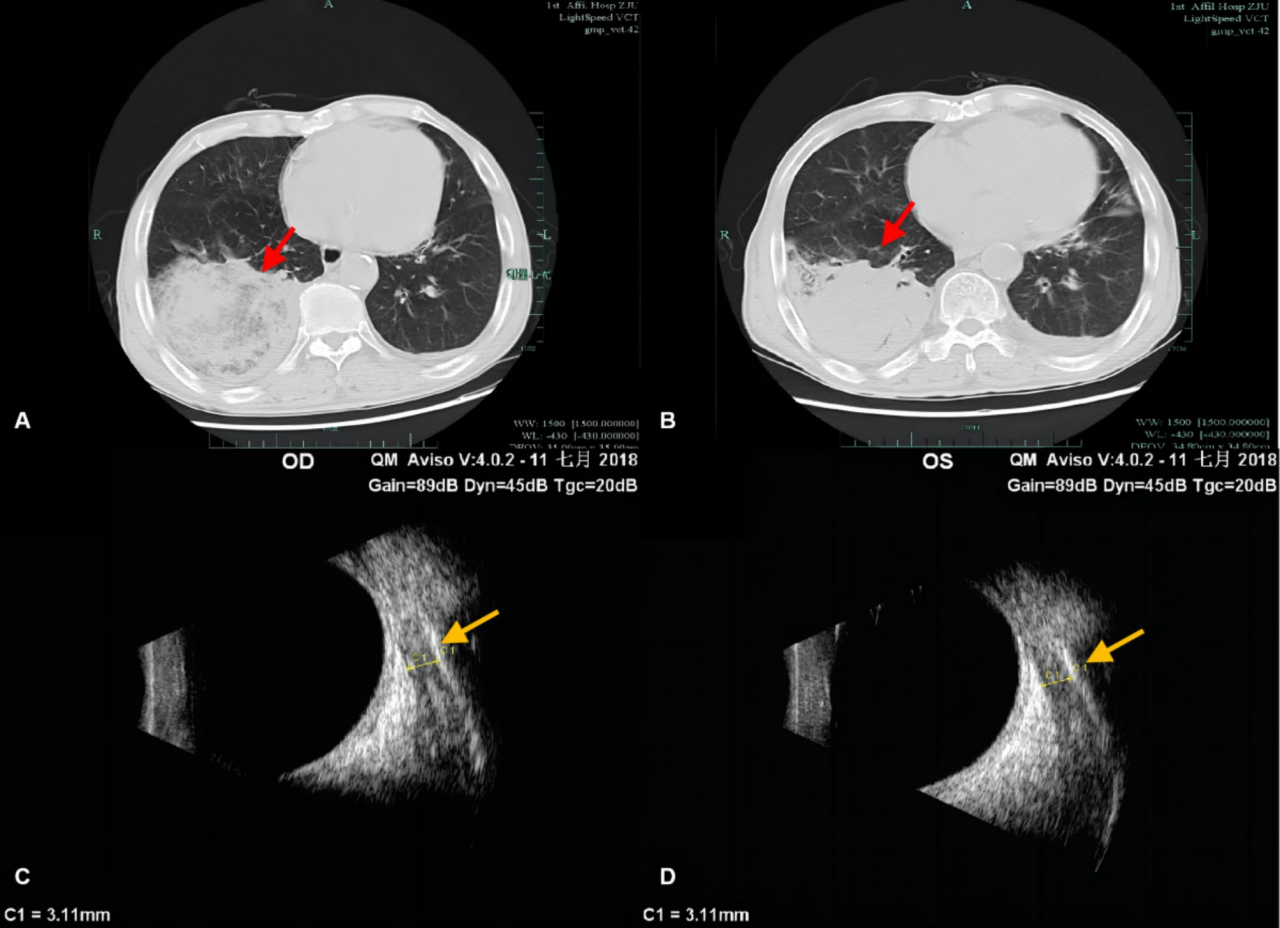
Lung CT and ocular ultrasound of case 3 **(A)** showing scattered infectious lesions in both lungs and a large patchy consolidation in the right lower lung when the fever occurred (red arrow). **(B)** Decreased lung inflammation and less extensive consolidation in the right lower lung 1 week later (red arrow). (**C**, **D**) Bilateral enlarged extraocular muscles due to TAO (yellow arrow)

A 79-year-old male was admitted to the Department of Ophthalmology for corticosteroid impulse therapy for thyroid-related ophthalmopathy (TAO). On admission, his VA was limited to FC because of bilateral exposure keratitis. The intraocular pressure (IOP) was 14 and 13 mmHg in the left and right eyes, respectively. The patient had a history of hypertension but was not taking any medication for it. The day after admission, both his eyelids were partially sutured for corneal protection. After hospitalization, he was administered 500 mg of intravenous methylprednisolone (MP) once daily for 5 days, which was tapered to 240 mg on the 6th day. On the evening of the 6th day, the patient developed a high fever (up to 40.3 °C) with cough, sputum, and coarse breath sounds. Lung CT showed scattered infectious lesions in both lungs and consolidation in the right lower lung (Fig. 3A). Blood and sputum were sent for culture prior to administering cefoxitin (CFX) intravenously. The next day, he developed redness and tenderness in both eyelids. Ocular B-scan revealed bilateral cataracts, vitreous opacities, and enlarged extraocular muscles (EOM) due to TAO (Fig. 3C,D); however, post-septal infection was not present.

He was then transferred to the infection unit. The sputum smear showed numerous gram-negative bacilli; hence, the antibiotic was changed to imipenem (IMP). The next day, blood and sputum cultures were shown to be positive for *Klebsiella. pneumoniae (K. pneumoniae)*, which is sensitive to IMP. Other treatments included expectorant administration, intravenous immunoglobulin and triglycerides for nutritional support, and ocular antibiotic eye drops and lubricating ointment. The infection gradually resolved, and he was discharged to a community hospital after 7 days of stay in the Infection ward. At discharge, both eyelids remained semi-sutured, the eyelid redness and swelling had subsided significantly, and the cough and sputum were relieved. Lung CT showed a reduction in inflammation and lesion consolidation (Fig. 3B). No recurrence of systemic or ocular infection was noted during the 7-month follow-up. At the last follow-up, there was no eyelid erythema or tenderness, no lagophthalmos, and the proptosis had regressed compared to that before steroid therapy. The ocular motility (OM) was restored, and IOP was 14 mmHg in both eyes. VA was limited to 20/50 in the right eye and 20/40 in the left due to bilateral corneal nebula formation and cataract.

### Case 4

A 30-year-old male with an abnormal white blood cell (WBC) count (28.7 × 10^9^/L) on physical examination (PE) was admitted to a local hospital and later diagnosed with acute myeloid leukemia (AML). His condition deteriorated quickly, with a high fever (up to 38.6 °C), pneumonia, splenomegaly, and pelvic, pleural, and pericardial effusion. Then, he was transferred to our facility for chemotherapy. Upon admission, a blood culture was performed and an anti-infective, anti-inflammatory, and diuretic therapy was initiated. Chemotherapy comprising the CIA regimen (cladribine 8.8 mg, days 1–3; idarubicin 20 mg, days 1–2 and 10 mg, day 3; cytarabine 170 mg, days 1–3 and 100 mg, days 4–5) was initiated on the 6th day of admission when the fever abated to 37.4 °C. The fever (38.6 °C) returned on the 2nd day of the chemotherapy course, and anal ulcers appeared on the 4th day. The patient experienced a critical myelosuppressive phase with persistent fever (37.3–38.9 °C) during and after the chemotherapy. Owing to negative blood culture results, a blood sample was sent for next-generation sequencing (NGS), which identified a mixed infection of *Enterococcus faecalis* (*E. faecalis)* and *Rhizopus oryzae* (*R. oryzaei)* 48 h later. NGS was performed twice to avoid interpretation errors.

**Fig. 4:**
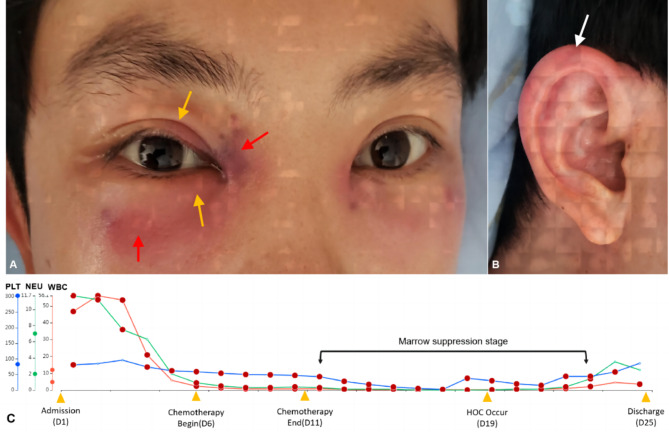
External eye and ear photographs when HOC occurred and blood counts during hospitalization of case 4. **(A)** Bilateral erythema of the eyelids (yellow arrows) and subcutaneous hemorrhage (red arrows) along with obvious tenderness. The symptoms on the left were milder than those on the right. **(B)** The auricle showed erythema and tenderness (white arrow). **(C)** White blood cell, neutrophil, and platelet count changes during the patient’s hospitalization along with important time points, including hospital admission and discharge, chemotherapy course, and HOC onset

On the 8th day after chemotherapy, the patient’s fever spiked to 39.9 °C, and he complained of redness and pain in both eyelids. A bedside OE revealed VA better than FC at 1 m in both eyes, with bilateral erythema, cutaneous ecchymosis, and eyelid tenderness. However, the conjunctiva, corneas, and lenses were unremarkable (Fig. 4A). Both fundi appeared myopic, with bleeding in the right retina without macular involvement. OM was unaffected, and no intraocular infection was noted. Bilateral auricular swelling and tenderness were present (Fig. 4B). As a result, bilateral pre-septal OC was considered, and topical ofloxacin ointment was applied. Due to the patient’s persistent septicemia and delayed pathogen results, his systemic antibiotic course was complicated. Initially, intravenous cefuroxime (CXM) was administered for 8 days, followed by intravenous MEM for 5 days, which was then switched to cefoperazone (CFP) for 12 days for targeting gram-negative microorganisms when the NGS identified *E. faecalis*. Next, intravenous VAN was given for 10 days, followed by tigecycline (TGC) for 6 days to target potential gram-positive organisms; prophylactic oral posaconazole (POS) was prescribed for 3 days, and intravenous voriconazole (VRC) for 6 days before NGS confirmed *R. oryzae* infection, and then, the medication was switched to intravenous liposomal amphotericin B (L-AMB) for 7 days with daily increments. Finally, the patient was prescribed oral POS before discharge, which he continued for approximately 5 months.

The patient’s systemic infection resolved, and HOC resolved after 6 days of onset. He was discharged on the 14th day after chemotherapy after a 25-day hospital stay (Fig. 4C). During the 6-month follow-up, he reported occasional fevers but no HOC recurrence. The best corrected VA (BCVA) was 20/25 in both eyes, although retinal hemorrhage persisted in the right fundus. He is still being closely monitored and treated for AML by the Hematology Department.

## Discussion and conclusions

OC is mostly caused by a direct spread of nearby infection and is more likely to occur in children than in adults; additionally, OC caused by septicemia is relatively rare [1, 4]. Ocular involvement in septicemia is mostly presented as endophthalmitis or panophthalmitis, and if OC is present, it is typically accompanied by panophthalmitis. HOC, which is an ocular complication of septicemia with only orbital involvement without intraocular infection, is extremely rare, and no case series on HOC have been reported previously.

For non-hematogenous OC or continuous spread OC, the most prevalent causative organisms are *Staphylococcus aureus*, *Streptococcus pneumoniae*, and *Haemophilus influenzae*, and odontogenic infections causing OC may also involve anaerobic bacteria [5]. The five causative pathogens identified in our case series are not commonly seen in OC. Case 2 acquired MRSA septicemia after squeezing a pimple. MRSA is the most prevalent pathogen causing skin infections in children; however, OC is a rare manifestation of MRSA infection. According to PH Blomquist’s study, only 1.3% of MRSA patients present with ocular involvement, and OC accounts for 19% of these cases [6]. Cases 1, 3, and 4 were infected with *C. albicans*, *K. pneumoniae, E. faecalis, and R. oryzae*, which are even rarer pathogens of OC compared to MRSA. To the best of our knowledge, only three cases of *C. albicans*-related OC have been published, including one case of panophthalmitis due to *C. albicans* septicemia and the other two due to non-hematogenous OC [7–9]. Additionally, no reports of OC caused by *C. albicans* septicemia without intraocular involvement have been published. *K. pneumoniae* is a highly invasive bacterium with the propensity to cause metastatic septic lesions with the eye being one of its target organs. In fact, *K. pneumoniae* is one of the most common causative organisms of nosocomial infections and endogenous endophthalmitis in China [10, 11]. The most prevalent form of *K. pneumoniae*-associated OC is *K. pneumoniae* panophthalmitis, including endogenous and exogenous panophthalmitis [12]. We noted eight published cases of *K. pneumoniae*-associated OC without intraocular involvement, and only two were due to hematogenous spread and both were post-septal OC [13, 14], which is different from our case who had pre-septal OC induced by hospital-acquired *K. pneumoniae* septicemia after glucocorticoids treatment. The prognosis of our case was much better compared to most ocular *K. pneumoniae* infections due to the absence of intraocular infection, though the BCVA of case 3 was limited to 20/40 and 20/50 because of TAO and cataracts.

Case 4 involved a combination infection caused by a bacterium and a fungus. Though his blood culture was negative, NGS identified *E. faecalis* and *R. oryzae* as the causative organisms. We found five cases of *E. faecalis*-associated OC reported in the literature, three of which were of endogenous panophthalmitis with intraocular infection, and the other two were of OC without intraocular involvement; however, as described by Biedner et al. in 1986, only one of these was due to septicemia, which was that of a female infant who presented with septicemia, ethmoiditis, and OC [15]. Another case of OC without intraocular involvement was that of a healthy 10-year-old Indian boy who developed OC from a contaminated hand after scratching his boils [16]. Another pathogen in case 4 was *R. oryzae*, a widespread saprophytic fungus that rarely affects healthy individuals, although it may cause infection in immunocompromised individuals and develop into mucormycosis. Invasive mucormycosis is an extremely aggressive fungal disease with a mortality rate of 22–59% [17, 18] and has become the third most common invasive fungal infection in adult patients with malignant hematologic diseases, such as in case 4 [19, 20]. Nearly 60% of mucormycosis is caused by *R. oryzae*, 90% of which are of the rhinio-orbital-cerebral mucormycosis (ROCM) type [21]. OC caused by *R. oryzae* usually presents as an ROCM phenotype with critical conditions and a dismal prognosis. Since case 4 only presented with pre-septal OC and auriculitis and did not show other typical ROCM manifestations, we speculate that the OC in case 4 might only be related to *E. faecalis*.

In our case series, the positivity rate of causative organisms was 100%, and the blood culture positive rate was 75% (3/4), which is clearly higher than that of non-bloodborne OC, which is approximately 2–7.9% [22, 23]. These findings are expected since the pathogens of HOC are transported via the bloodstream. Collecting blood or other samples for culture before administration of antibiotics may increase the positivity rate. When routine cultures are negative, NGS is recommended to identify the causative microorganism, as was done in case 4. NGS is a high-throughput sequencing method for massive parallel sequencing that uses targeted amplicon sequencing or microbial whole genome sequencing strategies to sequence millions of small DNA fragments simultaneously. NGS does not require sequence-specific amplification and is highly effective in diagnosing systemic or ocular infectious diseases of unknown origin or in patients with suspected infection who have negative culture results [24, 25]. NGS provides faster results, especially when the pathogen is likely to be a fungus, as most pathogenic fungi take 48–72 h and sometimes 2–4 weeks to grow. Contrarily, NGS can provide results in 48 h regardless of whether the organism is a bacteria, fungus, or parasite.

Patients with HOC are usually at high risk of other diseases due to septicemia. Early identification of pathogens and administration of targeted antibiotics to control systemic and ocular infections swiftly are essential for saving the patient’s life and vision. Case 4 had AML and thus belonged to a high-risk group of nosocomial fungal infections. Despite prophylactic administration of POS on admission, he experienced persistent fever during his hospitalization, especially in the post-chemo myelosuppression phase. When *R. oryzae* was identified by NGS, the antifungal medication was switched to L-AMB, and the infection was predictably controlled. Therefore, NGS is recommended to identify pathogens when patients are in critical condition and have negative culture or a poor response to empirical therapies. However, it should be acknowledged that the high cost of NGS testing, non-coverage by medical insurance, and bias in the interpretation of its results limit its widespread clinical use [24].

The vulnerable groups and the pathogenic spectrum of HOC differ from non-hematogenous OC, which often affects children and occasionally adults, usually immunocompetent, whereas the patients’ age in HOC varies widely, ranging from adolescents to the elderly, mainly affecting immunocompromised individuals, and the infection may be nosocomial, all of which share some similarities with endogenous endophthalmitis [26, 27]. In our case series, three of the four patients were immunosuppressed. Cases 1 and 3 were associated with steroid use, while case 4 had AML and underwent chemotherapy. The causative microorganisms in these three patients were *C. albicans*, *K. pneumoniae, E. faecalis, and R. oryzae.*, all of which are opportunistic pathogens and causative agents of nosocomial infections and have low chances of causing infection in immunocompetent individuals, thus seldom causing non-hematogenous OC.

The ocular manifestations of HOC are similar to those of non-hematologic OC and include eyelids erythema and periorbital tenderness; additionally, proptosis and restricted and painful OM is seen when the post-septal orbit is involved and is sometimes accompanied by decreased visual acuity. Patients with HOC may present with severe general conditions, such as persistent high fever, markedly elevated inflammatory indicators, and longer hospital stays. Three of the four HOC patients had a temperature > 39 ℃, with case 3 developing a maximum temperature of 40.3 ℃. WBC, neutrophils, and other inflammatory markers, such as c-reactive protein, erythrocyte sedimentation rate, and procalcitonin levels were significantly elevated in all cases. Among them, the WBC count of case 4 was mainly determined by his underlying disease and treatment rather than inflammation. The hospitalization durations of our cases ranged from 13 to 43 days, with an average of 24 days, much longer than that of non-blood-borne OC [1]. Septicemia is common in both HOC and non-hematogenous OC; in HOC, septicemia is the cause, whereas in non-hematogenous OC, septicemia is its complication. Both HOC and non-hematogenous OC have a high proportion of intracranial extension, with the former associated with microorganisms entering via hematogenous spread and the latter typically originating from direct infection extension from nearby structures. Typical clinical manifestations of brain abscess include headache, fever, and focal neurological deficits, but < 50% of cases have all triads [28]. Herein, two of the four patients had CNS involvement, as indicated by headache, neck stiffness, intracranial hypertension, imaging findings, and CSF test results. Sometimes microbes may be isolated from CSF samples, as seen in case 2.

HOC treatments are divided into three categories: anti-infection, systemic support, and ocular treatments, and infection control is key in its management. Due to the markedly different spectrum of the causative organisms, the experience with non-hematogenous OC may not be applicable for HOC, which is commonly caused by opportunistic pathogens and can be hospital-acquired, necessitating expert consultation and higher-level antibiotics, such as VAN; third- and fourth-generation cephalosporins; carbapenems; newly synthesized antibiotics; such as LZD, DAP, TGC; and a combination of antifungal agents in some cases [29]. Antibiotic regimens should be dynamically adjusted to the patient’s response, such as temperature, inflammatory factor levels, and culture results of the causative organism. HOC patients usually present with systemic diseases or an immunosuppressed state and are more likely to be in critical condition, thus requiring multidisciplinary and supportive treatments. The management of case 1, for example, required the involvement of the Emergency, Respiratory, Dermatology, Ophthalmology, Otorhinolaryngology, Infection, and Neurology Departments. As for the ocular treatments in OC, the requirement for surgical intervention varies from case to case. No surgical intervention is needed for pre-septal OC; however, in post-septal OC, the need for surgical intervention depends on the presence of an abscess and response to antibiotic therapy. When compared with non-hematogenous OC, which has a higher rate of abscess formation than HOC, HOC is less likely to lead to an orbital abscess and responds well to antibiotics, lowering the chance of surgical intervention [1, 30]. Besides ocular severity and response to anti-infective treatments, other factors affecting surgical intervention include imaging findings and the availability of orbital surgeons, otolaryngologists, and, in the case of pediatric patients, anesthesiologists [1, 30]. In our case series, only case 2 had an orbital abscess; nevertheless, the patient responded well to antibiotic treatment and showed gradual resolution of the abscess; thus, surgical intervention was spared. Other ocular treatments for OC included eye drops or ointments and treatment for comorbid ocular conditions unrelated to OC, such as TAO in case 3 and retinal hemorrhage in case 4.

Patients with OC suffer from restricted and painful eye movement, indicating the involvement of EOMs. In contiguously spreading OC, the medial rectus, which is closest to the sinuses, is usually the first and the most frequently involved EOM, [30, 31] while in HOC, it appears to be less frequently involved due to the different infection routes. In HOC, the EOM palsy may be induced by intracranial conditions affecting the innervating nerves. The adductor nerve has the longest pathway at the base of the skull and is most likely to be affected by intracranial lesions. Cases 1 and 2 were both of post-septal OC with intracranial hypertension, the lateral and superior recti were mainly affected, and the lateral rectus palsy was the last to recover; case 2 required 18 months for recovery.

Despite the relatively aggressive systemic condition, the ocular prognosis of HOC seems satisfactory. As for our case series, there are three main reasons for the good outcome. First, we excluded OC associated with panophthalmitis. No patients had any intraocular infection, thus presenting with a good VA at admission and post-treatment. Second, three of the four cases had no orbital abscess, with little impact on the eyeball and optic nerve, guaranteeing a good prognosis. Third, a satisfactory anti-infective effect on the OC resulted from the high culture-positive rate and the pathogen-targeted antibiotic regimen.

In conclusion, this is the first case series report of HOC that is free of intraocular involvement. HOC is a rare ocular complication of septicemia that can affect people of all ages, especially immunosuppressed adults. Compared to non-hematogenous OC, HOC has a more severe systemic presentation but a relatively milder ocular manifestation. Blood cultures are highly positive and NGS testing is recommended if routine cultures are negative. The causative microorganisms of HOC are predominantly opportunistic pathogens, possibly hospital-acquired and fungal. Infection control with systemic antibiotics based on the causative organism in combination with multidisciplinary management guarantees a favorable prognosis. This study will provide some guidance for future diagnosis and treatment of the ocular complications of septicemia.

## Data Availability

All data generated or analysed during this study are included in this published article.

## List of abbreviations

AM: Amoxicillin
AML: Acute myeloid leukemia
BCVA: Best corrected visual acuity
CAZ: Ceftazidime
CFP: Cefoperazone
CFX: Cefoxitin
CNS: Central Nervous System
CRP: C-reactive protein
CSF: Cerebrospinal fluid
CT: Computerized tomography
CXM: Cefuroxime
DAP: Daptomycin
EOM: Extraocular muscles
ESR: Erythrocyte Sedimentation Rate
FC: Finger counting
FCA: Fluconazole
GC: Glucocorticoids
HOC: Hematogenous orbital cellulitis
ICP: Intracranial pressure
IMP: Imipenem
IOP: Intraocular pressure
L-AMB: Liposomal amphotericin B
LP: Lumbar puncture
LZD: Linezolid
MEM: Meropenem
MP: Methylprednisolone
MRI: Magnetic Resonance Imaging
MRSA: *Methicillin-resistant Staphylococcus aureus*
MWGS: Microbial whole genome sequencing
MXF: Moxifloxacin
NGS: Next-generation sequencing
OC: Orbital cellulitis
OE: Ophthalmologic examination
OM: Ocular motility
PCT: Procalcitonin
PE: Physical examination
POS: Posaconazole
ROCM: Rhinio-orbital-cerebral mucormycosis
TAO: Thyroid-associated ophthalmopathy
TAS: Targeted amplicon sequencing
TGC: Tigecycline
TZP: Piperacillin-tazobactam
VA: Visual acuity
VAN: Vancomycin
VRC: Voriconazole
WBC: White blood cell
